# SARS-CoV-2 Saliva Mass Screening in Primary Schools: A 10-Week Sentinel Surveillance Study in Munich, Germany

**DOI:** 10.3390/diagnostics12010162

**Published:** 2022-01-11

**Authors:** Sebastian Vogel, Ulrich von Both, Elisabeth Nowak, Janina Ludwig, Alexandra Köhler, Noah Lee, Elisabeth Dick, Anita Rack-Hoch, Bernd Wicklein, Jessica Neusser, Tobias Wagner, Alexandra Schubö, Maxim Ustinov, Werner Schimana, Stephan Busche, Laura Kolberg, Martin Hoch

**Affiliations:** 1 Department Task Force Infectious Diseases, Bavarian Health and Food Safety Authority, Lazarettstrasse 67, 80636 Munich, Germany; Elisabeth.nowak95@googlemail.com (E.N.); Janina.ludwig@hotmail.com (J.L.); Alexandra.koehler@lgl.bayern.de (A.K.); Noah.lee@lgl.bayern.de (N.L.); Elisa.dick@web.de (E.D.); Bernd.wicklein@lgl.bayern.de (B.W.); Jessica.neusser@lgl.bayern.de (J.N.); Tobias.wagner@lgl.bayern.de (T.W.); Alexandra.schuboe@lgl.bayern.de (A.S.); Maxim.ustinov@lgl.bayern.de (M.U.); Martin.hoch@lgl.bayern.de (M.H.); 2 Dr. von Hauner Children’s Hospital, University Hospital, Ludwig-Maximilians-University, Lindwurmstrasse 4, 80337 Munich, Germany; Ulrich.von.both@med.lmu.de (U.v.B.); Anita.rack@med.uni-muenchen.de (A.R.-H.); Laura.kolberg@med.uni-muenchen.de (L.K.); 3 German Center for Infection Research (DZIF), Partner Site Munich, 80337 Munich, Germany; 4 Gesundheitsreferat Stadt München (GSR)/Public Health Department Munich, 80335 Munich, Germany; Werner.schimana@muenchen.de; 5 Eurofins LifeCodexx GmbH, 78467 Konstanz, Germany; S.Busche@lifecodexx.com

**Keywords:** SARS-CoV-2, saliva, mass screening, primary school, Salivette^®^, RT-qPCR

## Abstract

Representative, actively collected surveillance data on asymptomatic SARS-CoV-2 infections in primary schoolchildren remain scarce. We evaluated the feasibility of a saliva mass screening concept and assessed infectious activity in primary schools. During a 10-week period from 3 March to 21 May 2021, schoolchildren and staff from 17 primary schools in Munich participated in the sentinel surveillance, cohort study. Participants were tested using the Salivette^®^ system, testing was supervised by trained school staff, and samples were processed via reverse transcription quantitative polymerase chain reaction (RT-qPCR). We included 4433 participants: 3752 children (median age, 8 [range, 6–13] years; 1926 girls [51%]) and 681 staff members (median age, 41 [range, 14–71] years; 592 women [87%]). In total, 23,905 samples were processed (4640 from staff), with participants representing 8.3% of all primary schoolchildren in Munich. Only eight cases were detected: Five out of 3752 participating children (0.13%) and three out of 681 staff members (0.44%). There were no secondary cases. In conclusion, supervised Salivette^®^ self-sampling was feasible, reliable, and safe and thus constituted an ideal method for SARS-CoV-2 mass screenings in primary schoolchildren. Our findings suggest that infectious activity among asymptomatic primary schoolchildren and staff was low. Primary schools appear to continue to play a minor role in the spread of SARS-CoV-2 despite high community incidence rates.

## 1. Introduction

The role of young children and primary schools in the spread of the novel severe acute respiratory syndrome corona virus 2 (SARS-CoV-2) has been a focus of public debate. Due to an initial lack of evidence, scientists and policy makers recommended and implemented school closures to prevent the transmission of SARS-CoV-2. However, several studies provided evidence that young children in educational institutions are not primary drivers of the pandemic and that infectious activity in schools is rather associated with levels of community transmission [1,2,3,4]. Hence, closures of these facilities have only minor effects on the spread of SARS-CoV-2 [5,6] while affecting children’s and families’ well-being [7,8].

The United Nations (UNICEF) estimate that 168 million children worldwide have not attended school between March 2020 and February 2021 and that 214 million have missed at least 75% of classroom instruction time [9]. There have been warnings that the distress of the pandemic and school closures, in particular, may have severe short- and long-term negative effects on children’s physical and mental health [7,10]. Experts have observed an increase in depression symptoms [8], rising obesity rates [11,12], and a severe learning loss, with children from less educated families being disproportionally more affected [13].

At the same time, children usually do not suffer from severe clinical disease unless underlying conditions are present [14]. Reports on the SARS-CoV-2 Alpha variant (B.1.1.7) as well as recent data on the Delta variant (B.1.617.2), suggest no alteration in the severity of disease in children [15,16].

To assess the role of young children in the pandemic and to facilitate the safe operation of educational facilities, from June to November 2020 we investigated the feasibility of a SARS-CoV-2 surveillance tailored to primary schools, kindergartens, and nurseries [1]. While the integration of a testing scheme into the daily routine of these facilities was successful, the concept depended on a large team of medical staff, as samples were collected using oropharyngeal swabs. Consequently, this first feasibility study included a rather small sample size. To allow for larger sample sizes without additional medical staff, in an accompanying sub-study, we evaluated the Salivette^®^ saliva collection system for children aged 3 to 11 [17]. Accordingly, saliva has been shown to be a well-established diagnostic specimen for detecting SARS-CoV-2 RNA [18,19,20,21,22], a child-friendly sampling alternative to swab-based methods [23,24] and one potentially suitable for the mass screening of children [25]. The findings in our evaluation of the Salivette^®^ system confirmed that assessment [17].

The objective of this study was to assess the feasibility of saliva mass screening by implementing a saliva self-sampling concept for primary schoolchildren and staff. In addition, we set out to monitor infectious activity in primary schools during a time of high SARS-CoV-2 community incidence.

## 2. Materials and Methods

### 2.1. Study Design and Conduct

This was a prospective, uncontrolled, sentinel surveillance, cohort study on the incidence of asymptomatic SARS-CoV-2 infections as determined by RT-qPCR in primary schools. Between 3 March 2021 and 21 May 2021, we tested the saliva of students and staff of randomly selected public primary schools in Munich, Germany. Participants were recruited through participating schools’ principals and teaching staff, and an information video was created. Participation was voluntary. The study was analyzed and reported according to the Strengthening the Reporting of Observational Studies in Epidemiology (STROBE) reporting guideline (https://www.strobe-statement.org, last accessed on 21 December 2021).

### 2.2. Ethical Statement

The study protocol was approved by the ethics committee of the Ludwig-Maximilians-University Munich under project no. 21-0233. Written informed consent was obtained using an online registration and consenting platform meeting all data protection requirements as per European law.

### 2.3. Eligibility Criteria and Study Procedure

Students and staff were eligible for enrollment if they met the following criteria: (1) child (male or female) aged 5 to 13 years, (2) staff (male or female) without age limit, (3) child attending or staff working in the randomly selected primary school, (4) written consent obtained. Exclusion criteria were symptoms compatible with COVID-19, food or drink intake within 30 min prior to sampling, and incongruent or erroneous input of personal data into the registration portal.

Participants’ and/or their legal guardians’ consent was obtained during the online registration process on a multi-language registration website (www.muenchner-virenwaechter.de, last accessed on 21 December 2021). For each test, an individual QR/ID code was created. Selected staff members were trained during study initiation visits to supervise the sample collection. Staff members assigned the participants’ QR/ID code to a Salivette^®^ tube. Participants subsequently kept the Salivette^®^ absorbent cotton roll in their mouth for a minimum of two minutes before placing it in the tube and closing it with the lid. Participants then deposited the sample into a collection container. The registration and sampling process were contactless, avoiding any close-distance interaction between staff and participants and between participants. Before leaving the test site, disinfection of hands was performed. Participants received their results electronically (via text message and email) on the same day. Positive results were simultaneously reported to the Public Health Department Munich (GSR).

During the study period, school classes/groups were halved, and children usually attended in-classroom teaching on alternating days. In study week 5, compulsory nasal rapid antigen self-testing was introduced as a prerequisite for joining in-classroom teaching at primary schools in Munich. Students at schools participating in the study were free to choose saliva testing instead. The test frequency was determined by schools with a minimum of one test day per week. Regarding individual participation, no fixed test frequency was specified.

### 2.4. Laboratory Analysis

For saliva collection, the neutral Salivette^®^ with an absorbent cotton roll was used (SARSTEDT AG and Co KG, Nuembrecht, Germany; product number 51.1534). Salivette^®^ tubes were compatible with immediate processing in the laboratory.

The processing parameters were optimized during the study ramp-up phase until 1600× *g* for 2 min at room temperature was established. The mean saliva volume was assessed by measuring the processed and stored samples collected during study week 10, using a single channel pipette. After processing, samples were stored at 4 °C temperature for a maximum of 30 days before being disposed.

RNA was extracted using the GSD NovaPrime^®^ IVD RNA Extraction AE1 kit on KingFisher Flex 96 (Thermo Fisher Scientific, Waltham, MA, USA). RT-qPCR was carried out using the CE-IVD ViroBOAR MD v1.0 kit. An analysis of RNaseP was conducted to validate the presence of human sample material and to monitor the presence of RT-qPCR inhibitors. Each sample was analyzed according to the classification criteria outlined in Table 1 online.

Positive samples were analyzed by next-generation-sequencing (NGS) on Illumina instruments (2× 150 bp) using the NGS ARTIC protocol (Illumina, Inc., San Diego, CA, USA).

### 2.5. Statistical Analysis

Descriptive data are shown as median/mean, range and interquartile ranges (IQR) for continuous variables. Categorical variables are displayed as total and relative frequencies. The incidence in children and staff was compared using Fisher’s exact test. Positive results were graphically displayed in relation to time, 7-day incidence rates in the general population in the City of Munich and in the age group of 6 to 11 years. In addition, the distribution of specific SARS-CoV-2 variants during the study period was graphically displayed. Analyses were based on proprietary surveillance data by the Bavarian Health and Food Safety Authority. Calculations were performed in RStudio software, version 4.0.2, Python, version 3.7. and Excel 2016. Surveillance data was analyzed using SurvNet@RKI (German notification software; www.rki.de, last accessed on 21 December 2021).

### 2.6. Contact Tracing

Confirmed cases detected in the study were reported to local health authorities (GSR), which subsequently followed standard contact tracing protocols.

### 2.7. Online Questionnaire

At the end of study week 10, participating schools’ principals were asked to complete an online questionnaire on infection control measures in place at their primary school during the study period as well as an evaluation of the study concept. Online questionnaires were carried out using LimeSurvey, Version 3.22.22.

## 3. Results

### 3.1. Study Population

The study included 4433 participants (Table 1): 3752 children (median age, 8 [range, 6–13] years; 1926 girls [51%]) and 681 staff members (median age, 41 [range, 14–71] years; 592 women [87%]). Seventeen schools, representing 12.8% of primary schools, and 3752 children, representing 8.3% of public primary schoolchildren in Munich, participated in the study. The weekly number of participants, processed samples and test frequency are documented in Table 2.

### 3.2. Detection of SARS-CoV-2 Infection by RT-qPCR

**Figure 1:**
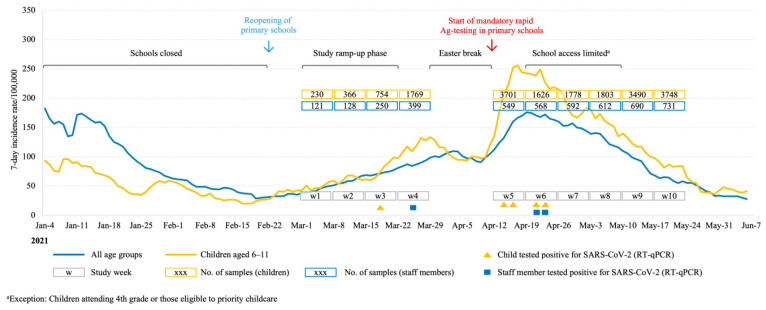
Pandemic activity and timeline of the Münchner Virenwächter 3.0 surveillance study in primary schools in the city of Munich, Germany, March–May 2021. Seven-day incidence rates were calculated in-house based on national surveillance data by the Bavarian Health and Food Safety Authority. RT-qPCR = reverse transcription quantitative polymerase chain reaction of saliva samples collected via the Salivette^®^. SARS-CoV-2 = severe acute respiratory syndrome coronavirus 2.

Out of 24,282 samples, 23,905 saliva specimens were processed, 19,265 from children and 4640 from staff (Table 2). A total of 377 samples were not suitable for processing due to an insufficient saliva volume (<300 μL), sampling errors, technical processing issues or misuse (Supplementary Figure S1). Over the 10-week study period, eight SARS-CoV-2 cases were detected (Figure 1). Five out of 3752 repeatedly screened children (0.13% [95% CI 0.06–0.31%]) and three out of 681 repeatedly screened staff members (0.44% [95% CI 0.15–1.29%]) tested positive on RT-qPCR. The comparison between the incidence in children and staff showed no statistically significant difference (*p* = 0.111). Cases were detected in weeks 3, 4, 5, and 6. In addition, we report three samples from children with very high Ct-values which, according to the study protocol, were classified as “inconclusive” (Supplementary Table S1). No secondary infections were detected.

### 3.3. Background Incidence

During the study period, the 7-day incidence rates in the general population in Munich ranged from 41 to 176 per 100,000 persons and from 43 to 252 per 100,000 persons in the age group of 6 to 11 years (Figure 1).

### 3.4. NGS and Distribution of SARS-CoV-2 Variants

Via NGS, four out of eight positive samples were classified as Alpha variant B.1.1.7, two as variant B.1., one as variant B.1.1, and one sample yielded no conclusive result. The Alpha variant accounted for an average of 62.1% of SARS-CoV-2 positive samples in Munich during the study period (Supplementary Figure S2).

### 3.5. Saliva Volume Measurement

The volume measurement of harvested saliva was conducted for 4515 samples, representing 18.6% of all study samples, and it showed a mean volume of 1554 μL (Table 3).

### 3.6. Infection Control Measure and Evaluation of Study Concept

At all participating schools, infection control measures were in place during the study period. Supplementary Table S2 contains a detailed overview of these measures. Supplementary Table S3 shows the results of the study evaluation by participating schools.

## 4. Discussion

In this large-scale, sentinel surveillance study, only eight out of 23,905 samples from 4433 participants in 17 primary schools tested positive for SARS-CoV-2. Five cases in 3752 children (0.13%) and three cases in 681 staff members (0.44%) were detected. Our findings are in line with other reports investigating infectious activity in primary schools [1,2,4,26]. However, when discussing the generalizability of these results, the individual study design, community incidence and dominant variants of concern (VOCs) at the time of investigation need to be considered. Ladhani and colleagues [4] reported that three out of 40,501 swabs from 11,966 participants had SARS-CoV-2 infection but covered three separate time periods over six months in 2020. Ismail and colleagues documented few SARS-CoV-2 infections in primary schools, especially when compared to other institutions like hospitals, care homes or other workplace settings [2]. We detected no secondary infections or outbreaks in participating schools. This may be attributed to the effectiveness of infection control measures in place during the study period and is in line with findings by Hershow and colleagues, who reported low secondary attack rates in primary schools while infection control measures, like wearing masks indoors, were in place [26]. Due to the emergence of new variants of concern, the findings summarized above need to be continuously re-evaluated by conducting further studies in educational facilities.

As previously described, the incidence of SARS-CoV-2 in primary schools has been associated with community incidence [1,2,3,4]. Prior to the introduction of mandatory testing, the incidence rates in children aged 6 to 11 years were lower than in the general population in Munich (Figure 1). Upon the return to schools in study week 5, following the Easter break and simultaneous with the start of mandatory testing, a peak in incidence in the age group of 6 to 11 years could be observed. During the same period, six out of a total of eight infections were detected in the study. We deduct that a large share of transmissions leading to these cases did not occur in a school setting, due to the incubation period of SARS-CoV-2, but could instead be traced back to private, family and household settings during the break in the weeks prior to detection. In addition, during this time, we observed a peak in community incidence.

Al Suwaidi and colleagues have described the advantages of saliva testing in school-age children [24]. In our study, we were able to safely and reliably collect and process 23,905 saliva samples from primary schoolchildren and staff. Thus, saliva testing proved to be feasible and suitable for mass screening, as suggested by Azzi [25].

A number of studies have monitored infectious activity in primary schools [1,2,4,26]. However, some were conducted prior to the emergence of VOCs or during periods of low community incidence or lockdown. Furthermore, suboptimal, unpleasant sampling methods like self-swabbing or nasopharyngeal swabs were used in children, and the sample size or test frequency was low. We addressed these limitations by implementing a saliva mass screening concept, recruiting 8.3% of Munich’s primary schoolchildren from 12.8% of the city’s primary schools and by processing a large number of tests. Importantly, this concept comprised a standardized, pre-analytic protocol, thereby avoiding inter- and intra-examiner variability, which may influence results obtained by swabbing. In addition, we monitored and confirmed the sampling quality by measuring the saliva volume in 18.6% of all study samples. Furthermore, the samples could be collected without a team of medical staff.

The study has some limitations: First, we cannot rule out false-negative results. Based on our experience from previous investigations (the sensitivity of the Salivette^®^ method in relation to oropharyngeal swabs was 94.9%), we consider this effect to be low [17,22]. Second, voluntary study participation may be associated with a selection bias, potentially over-representing participants with better adherence to infection control measures when in private or family settings. To mitigate this, we developed a straightforward, multi-language online registration process resulting in participation rates of up to 95% at participating schools. Third, when discussing the significance of our surveillance data, one needs to consider that, after a ramp-up phase in study weeks 1 to 3, larger sample sizes could be obtained from study weeks 4 to 10. Due to public health policy changes, school access during study weeks 6 to 8 was limited and participation of children temporarily dropped by >50%, in line with decreased attendance rates during that period (Table 2).

In summary, the test concept was very well-received by participants, guardians, and staff, and schools were able to integrate testing into their daily routine. We found saliva sampling using the Salivette^®^ to be simple, minimally invasive, and feasible for SARS-CoV-2 mass screening in children, especially when combined with a straightforward online registration process.

## 5. Conclusions

We conclude that, even during periods of high community transmission, asymptomatic infections in primary schools continue to play a minor role in the spread of SARS-CoV-2 when appropriate infection control measures are in place. In light of ongoing debates on how to operate primary schools in the pandemic, we recommend a continuous assessment of infectious activity. We are confident that saliva-based mass screening could play a relevant role in such surveillance activities.

## Figures and Tables

**Table 1:** Demographic characteristics of the study population. Abbreviations: IQR, interquartile range; y, years; No., number.

Characteristics	Participants, No. (%)	
	Staff	Children
Total No.	681	3752
Age, median,(range) [IQR], y	41 (14–71) [29–51]	8 (6–13) [7–9]
Sex		
Male	89 (0.13)	1826 (0.49)
Female	592 (0.87)	1926 (0.51)

**Table 2:** Participants, saliva samples and test frequency per study week. Data are presented as absolute numbers per study week.

Study Week	1	2	3	4	5	6	7	8	9	10
**Participants, No.**										
Total	341	485	965	2128	3194	1312	1381	1374	2705	2816
Children	230	363	741	1763	2808	961	1010	1005	2298	2403
Staff members	111	122	224	365	386	351	371	369	407	413
**Saliva samples, No.**										
Total	351	494	1004	2168	4250	2194	2370	2415	4180	4479
Children (all)	230	366	754	1769	3701	1626	1778	1803	3490	3748
Children (negative)	230	366	753	1769	3699	1623	1777	1802	3490	3748
Children (positive)	0	0	1	0	2	2	0	0	0	0
Children (inconclusive)	0	0	0	0	0	1	1	1	0	0
Staff members (all)	121	128	250	399	549	568	592	612	690	731
Staff members (negative)	121	128	250	398	549	566	592	612	690	731
Staff members (positive)	0	0	0	1	0	2	0	0	0	0
Staff members (inconclusive)	0	0	0	0	0	0	0	0	0	0
**Test frequency, No.**										
Children tested once	230	360	728	1757	1923	559	554	495	1258	1329
Children tested twice	0	3	13	6	878	179	197	258	910	843
Children tested three times	0	0	0	0	6	201	228	225	108	205
Children tested four times	0	0	0	0	1	4	9	18	22	12
Children tested five times	0	0	0	0	0	18	22	9	0	14
Staff members tested once	101	116	200	331	246	218	233	207	222	208
Staff members tested twice	10	6	22	34	120	74	78	103	107	127
Staff members tested three times	0	0	2	0	17	44	46	46	58	57
Staff members tested four times	0	0	0	0	3	5	5	6	20	7
Staff members tested five times	0	0	0	0	0	10	9	8	0	14

**Table 3:** Volume assessment of 4515 saliva samples from study week 10. Data are presented as the number of measured Salivette^®^ samples by age or age group.

Age Group [Years]	*n*	Mean [μL]	Min [μL]	Max [μL]
6	261	1495	300	3150
7	1045	1481	10	3600
8	916	1579	50	4300
9	889	1609	100	3900
10	608	1558	10	3600
11–13	60	1523	400	2750
6–13 (all children)	3779	1549	10	4300
14–71 (all staff)	736	1582	50	2650
All	4515	1554	10	4300

## Data Availability

The datasets generated and/or analyzed during this study are not publicly available due to the limitations of the participants’ consent forms.

## Supplementary Materials

The following supporting information can be downloaded at: https://www.mdpi.com/article/10.3390/diagnostics12010162/s1, Figure S1: Study sample flow, Figure S2: Distribution of SARS-CoV-2 variants per calendar week in the City of Munich, February to May 2021, Table S1: Classification of RT-qPCR laboratory test results, Table S2: Aggregated results of an online questionnaire on adherence to infection control measures in participating primary schools, Table S3: Aggregated results of an online questionnaire on the overall study concept evaluation.
