# Supplementary material for: SARS-CoV-2 Saliva Mass Screening in Primary Schools: A 10-Week Sentinel Surveillance Study in Munich, Germany
Source: Diagnostics (Basel). 2022 Jan 11;12(1):162. doi: 10.3390/diagnostics12010162 (PMC8774979; doi:10.3390/diagnostics12010162)
Supplement: Supplementary file 1 [file diagnostics-12-00162-s001.zip › supplementary_TableS2_11_25.pdf]

Supplementary Table S2: Aggregated results of an online questionnaire on adherence to infection control measures in participating primary schools. The survey was completed by each school's principal after study week 10. Survey participation rate was 100%. Absolute numbers and percentages (%) are displayed.

	**Number of primary schools (n=17)**
Primary schools adhering to infection control measures	17 (100%)
Supervision of children in half-size groups	12 (71%)
Physical distancing (> 1.5 m) between staff members inside school building	15 (88%)
Physical distancing (> 1.5 m) between staff members outside school building	13 (77%)
Physical distancing (> 1.5 m) between children inside school building	12 (71%)
Physical distancing (> 1.5 m) between children outside school building	12 (71%)
Face mask for staff members inside school building	17 (100%)
Face mask for staff members outside school building	17 (100%)
Face mask for staff members during drop-off/pick-up of children	16 (94%)
Face mask for parents during drop-off/pick-up of children	17 (100%)
Other protection measures (e.g. protection glass, face shield)	9 (53%)
Limited number of people/parents allowed on school premises when dropping off/picking up children	11 (65%)
Washing hands before dropping off children	8 (47%)
Separate use of bathroom facilities for individual groups/classes	8 (47%)
Closure of garden/playground areas/common rooms	2 (12%)
Separate use of garden/playground areas for individual groups/classes	15 (88%)
Handwashing before meals	16 (94%)
Handwashing before entering classes	16 (94%)
Hand disinfectant dispensers provided on premises	10 (59%)
Cancellation of common activities (parties, trips, bazaars etc.)	15 (88%)
Hygiene training for students and school staff	9 (53%)
