# Supplementary material for: SARS-CoV-2 Saliva Mass Screening in Primary Schools: A 10-Week Sentinel Surveillance Study in Munich, Germany
Source: Diagnostics (Basel). 2022 Jan 11;12(1):162. doi: 10.3390/diagnostics12010162 (PMC8774979; doi:10.3390/diagnostics12010162)
Supplement: Supplementary file 1 [file diagnostics-12-00162-s001.zip › supplementary-FigureS2_11_25.pdf]

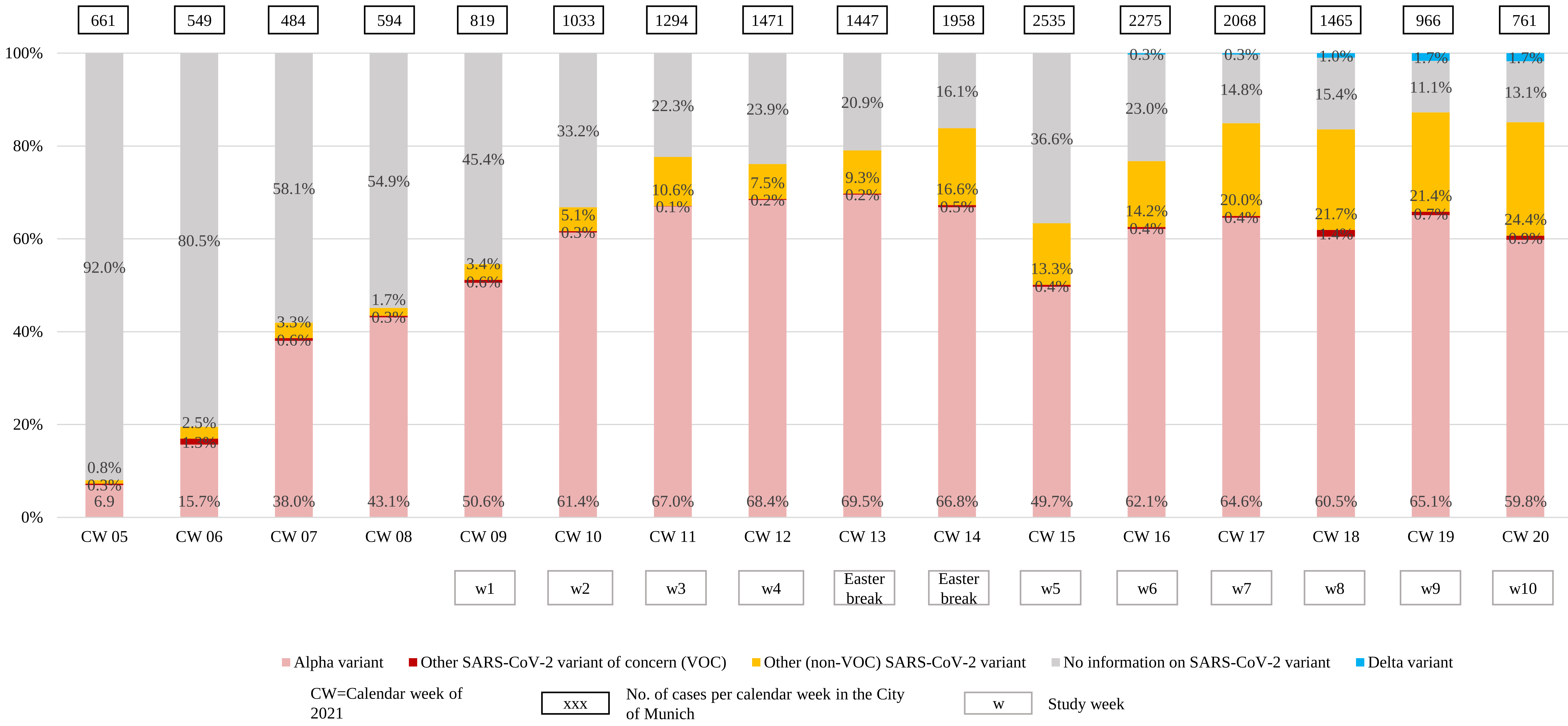

Supplementary Figure S2: Distribution of SARS-CoV-2 variants per calendar week in the City of Munich, February to May, 2021. Cases of Alpha variant (B.1.1.7) as well as other SARS-CoV-2 variants were defined as NGS-confirmed samples and variant-specific PCR-confirmed samples, where applicable. Other SARS-CoV-2 variants of concern include Beta variant (B.1.351), Delta variant (B.1.617.2), and Gamma variant (P.1). Other non-VOC variants e.g., include B.1.525, B.1.526. As of May 31, 2021, only variant B.1.617.2 (VOC Delta) was classified as a VOC by the World Health Organization (WHO). All data was analysed in-house based on national surveillance data (German Infection Protection act) by the Bavarian Health and Food Safety Authority.
