# Supplementary material for: SARS-CoV-2 Saliva Mass Screening in Primary Schools: A 10-Week Sentinel Surveillance Study in Munich, Germany
Source: Diagnostics (Basel). 2022 Jan 11;12(1):162. doi: 10.3390/diagnostics12010162 (PMC8774979; doi:10.3390/diagnostics12010162)
Supplement: Supplementary file 1 [file diagnostics-12-00162-s001.zip › supplementary-TableS1_11_25.pdf]

Supplementary Table S1: Classification of RT-qPCR laboratory test results. Abbreviations: N, Ct-value N-Gene; E, Ct-value E-Gene; NA, not applicable/not detected.

**Positive**	N ≤ 35 & E ≤ 33 or N ≤ 35 & E > 33 or N > 35 & E ≤ 33
**Inconclusive**	N > 35 & E > 33 or N ≤ 35 & E = NA or N = NA & E ≤ 33
**Negative**	N > 35 & E = NA or N = NA & E > 33 or N & E = NA
**Variant without distinction**	Mutations N501Y and A570D not determined
**Variant B.1.1.7**	Mutations N501Y and A570D determined
**Variant B.1.351 or B.1.1.28**	Mutation N501Y determined
**Variant inconclusive**	Positive classification based on N and E without clear determination of variant
**Invalid**	No signal in internal PCR control/insufficient saliva/technical error
