# Supplementary material for: SARS-CoV-2 Saliva Mass Screening in Primary Schools: A 10-Week Sentinel Surveillance Study in Munich, Germany
Source: Diagnostics (Basel). 2022 Jan 11;12(1):162. doi: 10.3390/diagnostics12010162 (PMC8774979; doi:10.3390/diagnostics12010162)
Supplement: Supplementary file 1 [file diagnostics-12-00162-s001.zip › supplementary-TableS3_11_25.pdf]

Supplementary Table S3: Aggregated results of an online questionnaire on overall study concept evaluation. The survey was completed by each school's principal after study week 10. Survey participation rate was 100%. Absolute numbers and percentages (%) are displayed.

	**Number of primary schools (n=17)**
Salivette-testing influenced the feeling of safety as follows: The feeling of safety was …	
5 – very much increased	8 (47%)
4 – much increased	7 (41%)
3 – increased	2 (12%)
2 – unchanged	
1 – decreased	
On a scale from 1 ("very bad") to 5 ("very good") I would rate the test concept and the handling of the Salivettes as follows:	
5 – very good	14 (82%)
4 – good	3 (18%)
3 – indifferent	
2 – bad	
1 – very bad	
In school grades I would rate the test concept and the handling of the Salivettes as follows:	
5 – very good	15 (88%)
4 – good	2 (12%)
3 – satisfactory	
4 – sufficient	
5 – poor	
6 – insufficient	
In my opinion students in 1st and 2nd grade are …	
very able to perform supervised self-tests with Salivettes	16 (94%)
able to perform testing with Salivettes	1 (6%)
partially able to perform testing with Salivettes	
rather unable to perform testing with Salivettes	
mostly unable to perform testing with Salivettes	
In my opinion students in 3rd and 4th grade are …	
very able to perform self-testing with Salivettes	17 (100%)
able to perform testing with Salivettes	
partially able to perform testing with Salivettes	
rather unable to perform testing with Salivettes	
mostly unable to perform testing with Salivettes	
I would rate the *Virenwächter 3.0* test concept (i.e. instruction of voluntary school staff members, online registration of samples at school etc.) as follows:	
Very good	13 (77%)
Good	3 (18%)
Indifferent	1 (6%)
Bad	
Very bad	
The study information and online registration website were translated into several languages during the course of the study. I think the translation into several languages was…	
extremely important	10 (59%)
very important	5 (29%)
rather important	1 (6%)
somewhat important	
not important at all.	1 (6%)
To increase voluntary participation rate, the electronic notification of test results (sample tracking message after laboratory arrival, text message and email with personalized test result) was …	

	Number of primary schools (n=17)
extremely important	10 (59%)
very important	5 (29%)
rather important	1 (6%)
somewhat important	
not important at all	1 (6%)
I wish to continue with Salivette-testing in our primary school	
Yes	13 (77%)
No	
Don't know	4 (24%)
I believe that the Salivettes are an appropriate self-testing tool for home-testing (i.e. children perform Salivette testing at home under supervision of their parents and subsequently bring the Salivettes to school)	
Agree	8 (47%)
Rather agree	4 (24%)
Neither agree nor disagree	1 (6%)
Rather disagree	2 (12%)
Disagree	2 (12%)
Comments	
When comparing antigen rapid tests and Salivette tests I think that Salivette tests are …	
much better.	15 (88%)
somewhat better.	2 (12%)
neither better nor worse.	
somewhat worse.	
much worse.	
