# Supplementary figures and images for: SARS-CoV-2 Saliva Mass Screening in Primary Schools: A 10-Week Sentinel Surveillance Study in Munich, Germany
Source: Diagnostics (Basel). 2022 Jan 11;12(1):162. doi: 10.3390/diagnostics12010162 (PMC8774979; doi:10.3390/diagnostics12010162)

**Supplementary Figure S1: Study sample flow**

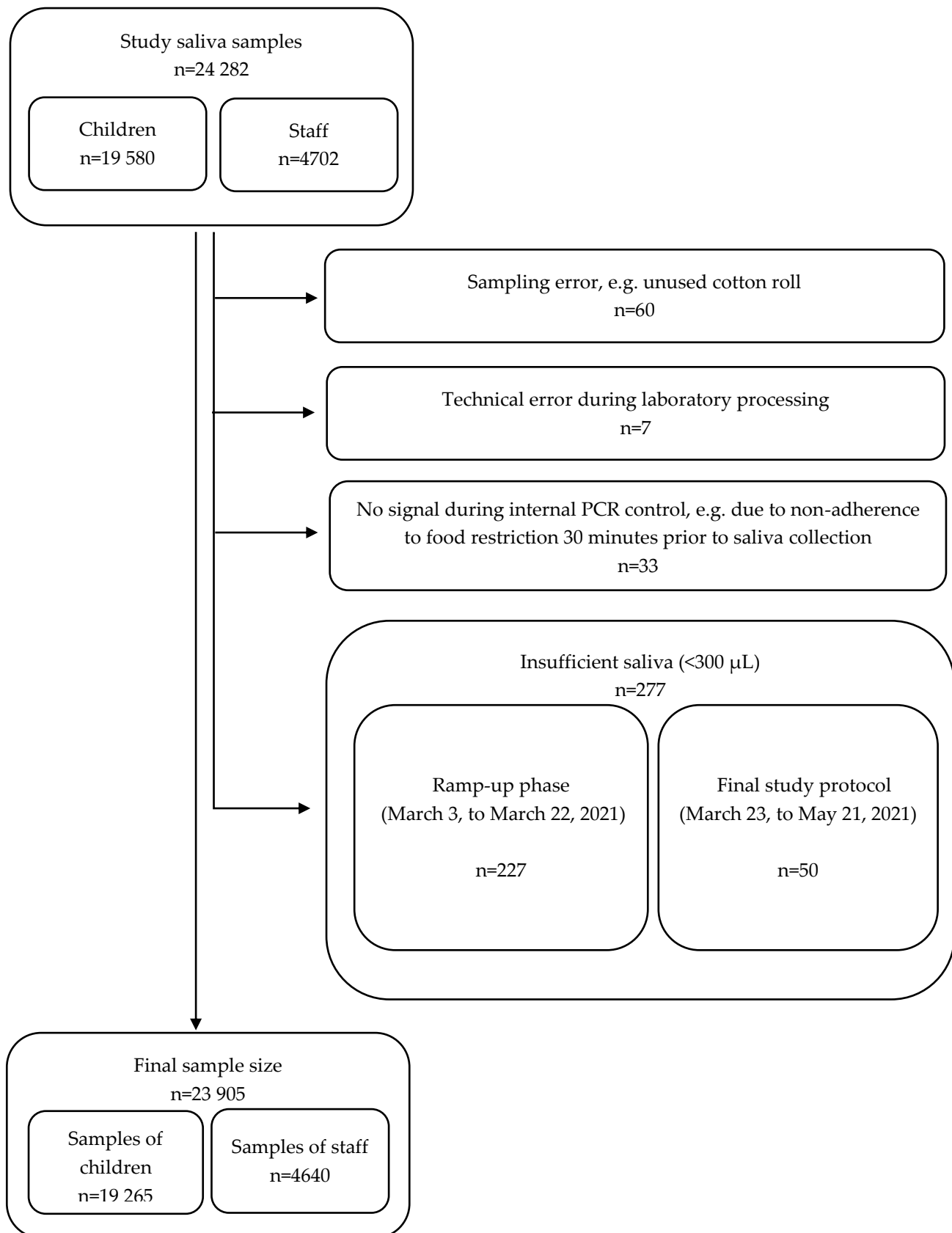

Supplement: Supplementary file 1 [file diagnostics-12-00162-s001.zip › supplementary-FigureS1_11_25.pdf]
